# Recover User’s Private Training Image Data by Gradient in Federated Learning

**DOI:** 10.3390/s22197157

**Published:** 2022-09-21

**Authors:** Haimei Gong, Liangjun Jiang, Xiaoyang Liu, Yuanqi Wang, Lei Wang, Ke Zhang

**Affiliations:** 1 College of Information and Communication Engineering, Hainan University, Haikou 570228, China; 2 Hainan Technology and Business College, Haikou 571100, China; 3 Funky-Tech (Shenzhen) Co., Ltd., Shenzhen 518000, China; 4 College of Automation, Chongqing University, Chongqing 400044, China

**Keywords:** security and privacy, Federated Learning, data reconstruction attack, gradient leakage attacks

## Abstract

Exchanging gradient is a widely used method in modern multinode machine learning system (e.g., distributed training, Federated Learning). Gradients and weights of model has been presumed to be safe to delivery. However, some studies have shown that gradient inversion technique can reconstruct the input images on the pixel level. In this study, we review the research work of data leakage by gradient inversion technique and categorize existing works into three groups: (i) Bias Attacks, (ii) Optimization-Based Attacks, and (iii) Linear Equation Solver Attacks. According to the characteristics of these algorithms, we propose one privacy attack system, i.e., Single-Sample Reconstruction Attack System (SSRAS). This system can carry out image reconstruction regardless of whether the label can be determined. It can extends gradient inversion attack from a fully connected layer with bias terms to attack a fully connected layer and convolutional neural network with or without bias terms. We also propose Improved R-GAP Alogrithm, which can utlize DLG algorithm to derive ground truth. Furthermore, we introduce Rank Analysis Index (RA-I) to measure the possible of whether the user’s raw image data can be reconstructed. This rank analysis derive virtual constraints $V_{i}$ from weights. Compared with the most representative attack algorithms, this reconstruction attack system can recover a user’s private training image with high fidelity and attack success rate. Experimental results also show the superiority of the attack system over some other state-of-the-art attack algorithms.

## 1. Introduction

In order to protect user’s privacy and meet law regulations, Google proposes Federated Learning in 2016. Currently, Federated Learning is widely used in health care [1,2], smart city [3], smart phone [4] and other fields. Although Federated Learning improves the privacy of local training data by exchanging model updates between clients and server, such as local gradients or updated parameters, and each client’s raw data is stored locally [4,5,6,7,8], some studies have shown that sharing even the local gradients update still have privacy risks. Adversary can make use of the updated gradients and weights to reconstruct the local training data [9,10,11]. Therefore, it is a worthy issue to study the influence of gradient on user’s private training image data disclosure.

**Figure 1:**
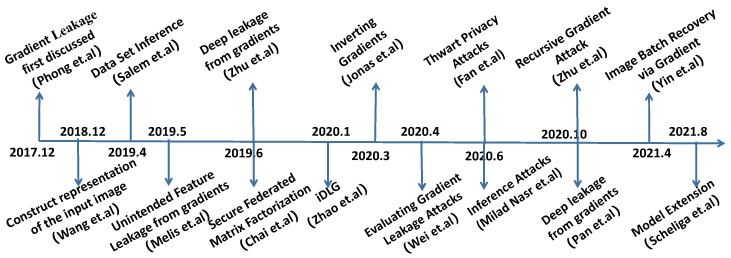
The history of gradient leakage.

Exchanging gradient is a widely used method in modern multinode machine learning system (e.g., distributed training, Federated Learning). It is meaningful to research the gradient’s safety. According to the research history of gradient leakage, we review relevant work, which is listed in Figure 1.

In 2017, Phong et al. [12] first show that user’s private training data can be recovered from gradient in theoretical. They make use of gradient of loss function about model weight parameters $\sigma_{k}$ and biases $\sigma_{b}$. From the Formula $\frac{\sigma_{k}}{\sigma_{b}}=\;x_{k}$, user’s private training data $x_{k}$ is completely leaked. This suggests a single-sample recovery is possible. Because of the use of biases, this type of attacks are called Bias Attacks.

Based on the conclude in [12] that gradient can compromise the privacy of user’s private training, Wang et al. [13] propose mGAN-AI attack against the federated learning for reconstructing private data of a specific victim. They first utilize an optimization approach to minimize the distance between gradients. This approach is adopted as a submodule in their Multitask GAN model. Their work first constructs a representation of the input image, then improves with a GAN. The optimization approach is the origin of the later Optimization-Based Attacks.

Salem et al. [14] aim at inferring different information of the updating set $D_{\mathrm{update}\;}$ in the output of a black-box Machine Learning model. δ denotes the posterior difference of both outputs. They propose a single-sample reconstruction attack $A_{SSR}$ to reconstruct the updating set itself. Extensive experiments show that full reconstruction in challenging conditions. However, the threat model in most distributed learning paradigms is not common.

Melis et al. [15] demonstrate with both CNNs and RNNs that periodical gradient updates during training can leak features as well as class memberships. Further, they also show that possible defences, such as selective gradient sharing, reducing dimensionality, and dropout are proved to be ineffective or have a negative impact on the quality of the collaboratively trained model.

Chai et al. [16] show server can deduce user’s rating data. However, there need knowing gradients of a user uploaded in two continuous steps. In the end, they enhance the distributed matrix factorization framework with homomorphic encryption.

Subsequently [13], Zhu et al. [9] officially propose Optimization-Based Attacks. They first randomly initialize a dummy input $x^{\prime }$ and label input $y^{\prime }$, then, feed these dummy data into models and get dummy gradient, at last, there need optimize the dummy gradient close as to original, which also makes the dummy data close to the real training data. They use L-BFGS [17] to perform the optimization. Experiments show the recovery from gradient is pixelwise accurate for images and tokenwise matching for texts, even if missing label information. Zhao et al. [10] extend the algorithm of [9] and show that label information can be computed analytically from the gradients of the last fully connected layer. They reconstruct the one-hot label of multiclass classification in terms of a single input.

Jonas Geiping et al. [11] adopt Optimization-Based Attacks, and follow the conclude of Bo Zhao et al. [10] who find the label information can be reconstructed analytically for classification tasks. Thus, they consider label information to be known. Experiments show that it is possible to faithfully reconstruct a image from parameter gradients. Further, They discuss several images does not protect the user’s privacy in federated learning applications.

Wei et al. [18] continue to work on Optimization-Based Attacks. They show the algorithm [9] is sensitive to initialization, and the same class image is an optimal initialization.

Fan et al. [19] analyze the Bias Attacks as a system of linear equations, and propose a method of perturbing the gradients to protect personal privacy. They propose a Secret Polarization Network (SPN). SPN consists of a public and a private network based on a backbone network. Fully connected polarization layers are kept private with its parameters not shared during the distributed learning process. Further, they first perform a rank analysis about network’s security.

Milad Nasr et al. [20] investigate the impact of gradients of different layers on attack accuracy, and show that the gradients of the later layers leak more membership information. It belongs to white-box inference attack. In [20], they argue that parameter aggregation in federated learning scenarios will have a negative impact on the accuracy of membership inference attacks. Furthermore, they propose to isolate target participant and improve the accuracy of attacker.

Pan et al. [21] show theory-oriented deep leakage from gradients via linear equation solver. The concept of Linear Equation Solver Attacks is first proposed. They analysize neural networks with ReLU activation function and use Optimization-Based Attacks technique to form a sparse linear equation system about gradients. In the process of solving the gradient equation, they made use of the tricky properties of ReLU. They focus their attacks on fully connected neural networks.

Zhu et al. [22] break the gradient attack down to a recursive process of solving systems of linear equations and propose recursive procedure to recover data from gradients in deep neural networks, which is named Recursive Gradient Attack. It belongs to Linear Equation Solver Attacks. They achieve an analytic gradient attack from fully connected layers to CNNs for the first time. Furthermore, they also propose a novel rank analysis to estimate the feasibility of performing gradient based privacy attacks.

Yin et al. [23] extend single image label restoration [10], and formulate label restoration algorithm for batch size K. They assume nonrepeating labels in batch. Furthermore, they adopt Optimization-Based Attacks technique to realize a full recovery of detailed individual images from batch averaged gradients in deep networks such as ResNet-50.

Scheliga et al. [24] introduce a PRivacy EnhanCing mODulE (PRECODE) that can be used as generic extension for arbitrary model architectures. It can be used for privacy leakage defense mechanisms.

In summary, the research history of gradient mainly focus on privacy attacks and privacy defense strategies. Existing works about privacy attacks can be roughly categorized into three groups: (i) Bias Attacks, bias reconstruction attacks mean to use gradients about bias term and weights to realize attack [12,19]. This attack can be solved by removing the bias term. (ii) Optimization-Based Attacks, the core idea of Optimization-Based gradient attacks are to minimize the distance between gradients. It first appears in [13], subsequently [9,10,11,18] refine the method. (iii) Linear Equation Solver Attacks, the main idea is to form gradient equation or weight equation, further, relizes reconstruct attacks [19,21,22]. In [19], they use a bias term and weights to form a gradient equation to realize attacks. It only studys a single neuron. While in [21], they rely on Optimization-Based gradient technique to form a gradient equation. In [22], they break the gradient attacks from fully layer to convolution layer, furthermore, make use of gradient constraints and weight constraints to form a matrix, solving the matrix can derive training image data.

Usually, there is a paradox between privacy reconstruction and privacy protection. Privacy-Preserving Deep Learning aims to collaboratively train and share a deep neural network model among multiple participants, without exposing their private training data. There are three common methods as privacy defense strategies: Differential-Privacy, Homomorphic Encryption, and Secure Multiparty Computation. In [25], differential privacy can be used for solving the privacy problem of crowdsourced. In [26], they focus on privacy leakage issue of publishing well-trained deep neural network models, differential privacy can be used for solving the problem. In [16], they enhance the distributed matrix factorization framework with homomorphic encryption. In [27], They provide formal data privacy guarantees using both differential privacy and secure multiparty computation frameworks. In order to proptct privacy, there are also some researchers to study Machine Learning Approaches for Malware Detection [28,29,30]. In this paper, our work mainly focus on gradient inversion attack algorithms.

In this article, our work mainly studies the most representative gradient inversion algorithms. DLG algorithm [9] belongs to Optimization-Based Attacks. It can recover private training data pixelwise accurate for images without label. However, this algorithm only can reconstruct image at the fully connected layer. Inverting Gradients [11] also belongs to Optimization-Based Attacks. It can faithfully reconstruct images at high resolution to fully connected layer. However, this algorithm can work under the premise of label information is known. R-GAP [22] belongs to Linear Equation Solver Attacks. It works as well as or even better than Optimization-Based Attacks. This algorithm extends attack from the fully connected layers to CNNs, However, this algorithm works under the premise of label information is known. Therefore, according to the characteristics of these algorithms, we propose one privacy attack system, i.e., Single-Sample Reconstruction Attack System (SSRAS). This system combines the advantages of most representative attack algorithms, which can realize image reconstruction regardless of whether the label can be determined. It can extends gradient inversion attack from a fully connected layer with bias terms to attack a fully connected layer and convolutional neural network with or without bias terms. Further, we propose Improved R-GAP Alogrithm, which can utlize DLG algorithm to derive ground truth. In short, compared with the most representative attack algorithms, this reconstruction attack system can recover user’s private training image with high fidelity and attack success rate. Experimental results also show the superiority of the attack system over some other state-of-the-art attack algorithm.

Our main contributions are as follows:

- We propose one privacy attack system, i.e., Single-Sample Reconstruction Attack System (SSRAS). This system combines the advantages of most representative attack algorithms, which can realize image reconstruction regardless of whether the label can be determined. It can extends gradient inversion attack from a fully connected layer with bias terms to attack a fully connected layer and convolutional neural network with or without bias terms.
- R-GAP works only if the label information is known. In this section, we propose Improved R-GAP Alogrithm, which can utlize DLG algorithm to derive ground truth. Further, it can extends the attack from the fully connected layer to the convolutional layer.
- We introduce Rank Analysis Index (RA-I) to measure the possible of whether the user’s raw image data can be reconstructed. This rank analysis derive virtual constraints $V_{i}$ from weights. This Rank Analysis Index(RA-I) is superior to [19].
- In order to guide attack from gradient towards natural images, we adopt $R_{\mathrm{fidelity}\;}\left(\cdot \right)$ to the loss function to steer reconstructed image away from unrealistic image. Improved R-GAP Alogrithm can make use of the difference of the reconstructed image, and adopt smoothed version.
- Simulation experiments and analysis of the optimization scheme verify that gradients encode a large amount of information, and this reconstruction attack system can recover user’s private training image with high fidelity and attack success rate, regardless of whether the label can be determined.

The rest of the paper is organized as follows: Section 2 describe the fundamental milestone framework of gradient leakage. Section 3 propose one privacy attack system, i.e., Single-Sample Reconstruction Attack System (SSRAS). Section 4 propose Improved R-GAP Alogrithm. Section 5 the experimental results are shown. Section 6 conclude the paper and give the further work.

## 2. Related Work

In this section, we review existing works about privacy attacks, and explain three types of attacks, for example Bias Attacks, Optimization-Based Attacks, and Linear Equation Solver Attacks.

### 2.1. Bias Attacks

In [12], Phong et al. first show that recover user’s private training data from gradient is possible. It is called Bias attacks. $x_{i}$ is the input data, $1\;\leq \;x_{i}\;\leq \;n$, *y* is a corresponding truth label, $w_{i}$ is the weight parameter to be learned, $1\;\leq \;w_{i}\;\leq \;n$, *b* is the bias, *f* is an activation function. The loss function is defined as the distance between the predicted value and the truth value. The predicted value is $h_{w,b(x)}=f\left(\sum_{i=1}^{i=n}w_{i}x_{i}+b\right)$, the truth value is *y*. The loss function

(1) $$\ell \left(w,b,x,y\right)\overset{\;\mathrm{def}\;}{=}{\left(h_{w,b}\left(x\right)-y\right)}^{2}$$

Gradient on the training sample is exactly the partial derivative of the loss function w.r.t.the model weight parameter and the bias.

(2) $$\sigma_{k}=\frac{\partial \ell (w,b,x,y)}{\partial w_{k}}=2\left(h_{w,b}\left(x\right)-y\right)f^{\prime }\left(\sum_{i=1}^{d}w_{i}x_{i}+b\right)\cdot x_{k}$$

(3) $$\sigma_{b}=\frac{\partial \ell (w,b,x,y)}{\partial b}=2\left(h_{w,b}\left(x\right)-y\right)f^{\prime }\left(\sum_{i=1}^{d}w_{i}x_{i}+b\right)$$

According to Formulas (2) and (3), we can have

(4) $$\frac{\sigma_{k}}{\sigma_{b}}=\;x_{k}$$

Therefore, from Formula (4), we can draw a conclude $x_{k}$ is completely leaked if the gradients are shared to server. In theory, this suggests a single-sample recovery is possible. However, we can simply disable this attack by removing the bias term. Besides, because of dimension mismatch, this way can not work on convolutional neural networks.

Fan et al. [19] aim at sloving the Bias Attacks. They propose a Secret Polarization Network(SPN), which is a method of perturbing gradients. The architecture of the SPN contains backbone network, and fully connected polarization layers. Thereof, fully connected polarization layers are devided into public and a private network. The parameters of private network are not shared in distributed learning. The loss about gradients $\nabla_{w,b}L$ can be shown as follows,

(5) $$\nabla w,bL=\alpha_{1}\cdot L_{CE}\left(u,y\right)+\underset{\text{secret perturbation}}{\underset{︸}{\alpha_{2}\cdot L_{P}\left(v,t\right)}}$$

$\alpha_{1}$ and $\alpha_{2}$ denote hyperparameters, $\alpha_{1}+\alpha_{2}=1$, $L_{CE}$ denotes cross-entropy loss, $L_{P}$ denotes polarization loss. The function of $L_{P}$ is to introduce interference, $\alpha_{2}$ controls the protection levels of training data. Fan et al. [19] also believe convolutional networks and fully connected networks are equivalent. However, they do not take into account that gradients are aggregated in a convolutional network. Further, they also first propose a rank analysis to estimate the security of network.

### 2.2. Optimization-Based Attacks

Wang et al. [13] propose mGAN-AI attack against the federated learning for reconstructing private data of a specific victim. They first utilize an optimization approach to minimize the distance between gradients. This approach is adopted as a submodule in their Multitask GAN model. Their work first constructs a representation of the input image, then improved with a GAN.

Subsequently [13], Zhu et al. [9] propose that even the absence of label information, the recovery from gradient is pixelwise accurate for image. Zhao et al. [10] extend the algorithm of [9] and show that label information can be computed analytically from the gradients of the last fully connected layer. They reconstruct the one-hot label of multiclass classification in terms of a single input.

The central recovery mechanism discussed in [9,10,23] is to recover the data from gradients. Zhu et al. [9] first randomly initialize a dummy input $x^{\prime }$ and label input $y^{\prime }$, then feed these dummy data into models and get dummy gradient, at last, they adopt L-BFGS [17] to perform the optimization. Zhao et al. [10] make use of the shared gradients of fully connected layer to extract the ground-truth label, then they can extract the data more effectively based on correct label. Yin et al. [23] extend single image label restoration [10], and formulate label restoration algorithm for batch size K. They assume nonrepeating labels in batch. Furthermore, they adopt Optimization-Based Attacks technique to realize a full recovery of detailed individual images from batch averaged gradients in deep networks, such as ResNet-50.

In [9,10], they all optimize the dummy gradients close to original gradients, which also make the dummy data close to the real training data. Their optimization adopts euclidean.

(6) $$\nabla W^{\prime }=\frac{\partial \ell \left(F\left(x^{\prime },W\right),y^{\prime }\right)}{\partial W}$$

(7) $$\begin{array}{cc} x^{\prime }{}^{*},y^{\prime }{}^{*} & =\underset{x^{\prime },y^{\prime }}{\arg\min}{∥\nabla W^{\prime }-\nabla W∥}^{2}\\ \; & =\underset{x^{\prime },y^{\prime }}{\arg\min}{∥\frac{\partial \ell \left(F\left(x^{\prime },W\right),y^{\prime }\right)}{\partial W}-\nabla W∥}^{2} \end{array}$$

The cost function is minimized to recover the original input image $x^{\prime }{}^{*}$,$y^{\prime }{}^{*}$ from transmitted gradient $\nabla_{\theta }L_{\theta }\left(x,y\right)$. Note that, this optimization requires 2^nd^ order derivatives.

Zhao et al. [10] firstly propose the ground truth label information can be derived from gradients of the last fully connected layer. They adopt a classification scenario, the loss function is defined as follows,

(8) $$l\left(x,c\right)=-\log\frac{e^{y_{c}}}{\Sigma_{j}e^{y_{j}}}$$

x denotes input data, c denotes corresponding ground-truth label, $y_{i}$ denotes the predicting score of the $i_{\mathrm{th}}$ class. The loss about output $y_{i}$ partial can derived gradients according to Formulas (9) and (10),

(9) $$g_{i}=\frac{\partial l(x,c)}{\partial y_{i}}=-\frac{\partial \log e^{y_{c}}-\partial \log\Sigma_{j}e^{y_{j}}}{\partial y_{i}}$$

(10) $$g_{i}=\left\{\begin{array}{cc} -1+\frac{e^{y_{i}}}{\sum je^{y_{i}}} & \;\mathrm{if}\;i=c\\ \frac{e^{y_{i}}}{\sum je^{y_{i}}} & \;\mathrm{else}\; \end{array}\right.$$

The gradient vector $\nabla W_{L}^{i}$ is the weight $W_{L}^{i}$ connected to the $i^{\mathrm{th}}$ logit in the output layer. Usually, the gradients of model weights are shared. Combining with Formula (10), there are

(11) $$\begin{array}{cc} \nabla W_{L}^{i} & =\frac{\partial l(x,c)}{\partial W_{L}^{i}}\\ \; & =\frac{\partial l(x,c)}{\partial y_{i}}\cdot \frac{\partial y_{i}}{\partial W_{L}^{i}}\\ \; & =g_{i}\cdot \frac{\partial \left(W_{L}^{i}{}^{T}a_{L-1}+b_{L}^{i}\right)}{\partial W_{L}^{i}}\\ \; & =g_{i}\cdot a_{L-1}, \end{array}$$

where the network has L layers, $y=a_{L}$ is the output of *L* layer, $b_{L}^{i}$ is the bias parameter of *L* layer, $y_{i}=W_{L}^{iT}a_{L-1}+b_{L}^{i}$.

Combining Formulas (10) and (11), the ground-truth label *c* can be predicted as follows,

(12) $$c=i,\;\;s.t.\;\;\nabla W_{L}^{i^{T}}\cdot \nabla W_{L}^{j}\leq 0,\;\forall j\neq i$$

The conclude of ground-truth *c* can be derived according to the sign of gradient. The signs of $\nabla W_{L}^{i}$ and $g_{i}$ are the same. The negative gradient can be the index of the ground-truth label. Note that, all the conclude need the assumption that there is non-negative activation function.

Jonas et al. [11] propose that it is no matter to architecture of training deep networks or trained deep networks, any input to a fully connected layer can be reconstructed. They consider that label information is known, this assumption base on the conclusion of Zhao et al. [10]. They adopt cosine similarity loss function, and add αTV(x) to control the image prior to the overall problem. Furthermore, they replace L-BFGS with Adam for optimization about networks, such as ReLU or LeakReLU. The specific form is defined as Formula (13),

(13) $$\arg\min_{x\in {\left[0,1\right]}^{n}}1-\frac{\langle \nabla_{\theta }L_{\theta }\left(x,y\right),\nabla_{\theta }L_{\theta }\left(x^{*},y\right)\rangle }{∥\nabla_{\theta }L_{\theta }\left(x,y\right)\left|∥|\nabla_{\theta }L_{\theta }\left(x^{*},y\right)∥\right.}+\alpha \mathrm{TV}\left(x\right)$$

In [11], they also discuss that federated average algorithm can average gradients over several iterations or several images in a batch, but it does not protect the user’s privacy in federated learning applications. They use a ConvNet architecture, which is roughly similar to AlexNet [31].

In [18,23], they propose to use L2 loss function to recover the original input image. Wei et al. [18] show that the algorithm of Zhu et al. [9] is sensitive to initialization, the optimal initialization is the same class image. The specific form is defined as Formula (14),

(14) $$\begin{array}{cc} x^{\prime }{}^{*},y^{\prime }{}^{*} & =\underset{x^{\prime },y^{\prime }}{\arg\min}{∥\nabla w_{att}^{\tau }\left(t\right)-\nabla w_{k}\left(t\right)∥}^{2}\\ \; & +\alpha {∥f\left(x_{rec}^{\tau },w\left(t\right)\right)-y_{rec}∥}^{2} \end{array}$$

$\nabla w_{k}\left(t\right)$ is the gradient of local training on private training data, $\left(x^{\prime },y^{\prime }\right)$ is attack seed, the gradient of attack seed is $\nabla w_{att}^{\tau }\left(t\right)$, $\left(x_{rec},y_{rec}\right)$ is reconstructed training data, α is regularizer ratio.

Pan et al. [21] show theory-oriented privacy analysis in neural networks with ReLU for data reconstruction attacks. They rely on Optimization-Based gradient technique to form gradient equations, The specific form is shown as follows,

(15) $$\sum_{i=1}^{M}\frac{\partial \ell \left(f\left(X_{i};W\right),Y_{i}\right)}{\partial W}=M\bar{G}$$

${\left\{\left(X_{i},Y_{i}\right)\right\}}_{i=1}^{M}$ are variables in this equation. In solving the gradient equation, they take advantage of ReLU’s properties.

### 2.3. Linear Equation Solver Attacks

In [19], they use a bias term and weights to form a gradient equation to realize attack. While, in [21], they rely on Optimization-Based gradient technique to form a gradient equation. In [22], they break the gradient attacks from fully layer to convolution layer, furthermore, make use of gradient constraints and weight constraints to form a matrix, solving the matrix can derive training image data. They also propose to estimate the feasibility of performing gradient attacks by rank analysis. The most representative attack is R-GAP [22]. The concrete form of gradient constraints can be described as Formula (16),

(16) $$K_{i}x_{i}=\mathrm{flatten}\left(\frac{\partial \ell }{\partial W_{i}}\right)$$

$x_{i}$ denotes the input in the ith layer, $K_{i}$ denotes coefficient matrix containing all gradient constraints in the ith layer.

The concrete form of weight constraints can be described as Formula (17). It needs assumption that they know the input of the subsequent layer.

(17) $$W_{i}x_{i}=Z_{i};\;Z_{i}\leftarrow f_{i}$$

$W_{i}$ represents convolutional kernel, $\left|Z_{i}\right|$ is weight constraints.

There is research about property inference attack in [32]. Researchers [32] explore to infer properties of training data using the model parameters. It belongs to retrieving input attributes from local updates, and is a shallow leak. Property inference can be derived directly in the reconstruction phase or by classifying the reconstructed data.

Some researchers propose model inversion attack. They show that the attacker attempts to obtain information of the training dataset from the trained model [13,33,34]. They utilize data representation to infer attribute values of data samples. With the development of Generative Adversarial Network, GAN-based reconstruction attack appear in [13]. The participant utilizes GAN structure to construct sensitive information about the victim. They can infer general image composition or dominating colors. Model inversion attack relies on a given layer and only reconstructs similar image. Fredrikson et al. [33] demonstrate that model inversion attack can recover similar image from facial recognition system. Pan et al. [34] utilize the intermediate data representation to infer sensitive attribute values of data samples. Model inversion attack generally is challenging for deeper neural network architectures if no additional information is provided.

Model extraction attack refers to an attacker trying to steal the parameters and hyperparameters of the model, Further, it can break model confidentiality or infer user data sets and model characteristics. In [35], they use model interpretation to reconstruct significant parts of training set.

## 3. SSRAS: Single-Sample Reconstruction Attack System

According to the research of related work, we have the pros and cons of the main gradients inversion algorithms. In this section, we propose one privacy attack system, i.e., Single-Sample Reconstruction Attack System (SSRAS). We first show threat model and attack objective. Then, we explain the key components of the SSRAS.

### 3.1. Threat Model and Attack Objective

Federated Learning improves the privacy of local training data by exchanging model updates, such as local gradients or updated parameters. Howerever, some attack algorithms have shown that the adversary can utilize gradients to obtain user’s private training image data. There are pros and cons of existing privacy attack algorithms. The most representative attack algorithms are listed in Table 1. The first attack Algorithm 1 (DLG) [9] and Algorithm 2 (Inverting Gradients) [11] belong to Optimization-Based Attacks from Gradients. Algorithm 3 (R-GAP) [22] belongs to Linear Equation Solver Attacks. DLG algorithm [9] belongs to Optimization-Based Attacks. It can recover private training data pixelwise accurate for images without label. However, this algorithm only can reconstruct image to fully connected layer. Inverting Gradients [11] also belongs to Optimization-Based Attacks. It can faithfully reconstruct images at high resolution to fully connected layer. However, this algorithm can work under the premise of label information is known. R-GAP [22] belongs to Linear Equation Solver Attacks.

Federated Learning trains a shared global model by a server and client working together. This security threat may come from: honest-but-curious server or malicious client. During training, server and client konw architecture of neural network, weights, gradients and other relevant information. Weight and gradient are transmitted in plaintext [4], instead of using privacy-preserving deep learning techniques [25,26,27,36], such as Differential-Privacy, Homomorphic Encryption, and Secure Multiparty Computation. The main purpose is to investigate whether user’s privacy security can be ensured by transmitting only gradients and weights in federated learning. Furthermore, it is necessary to study how to use gradients and weights to recover the private training image data of users.

Ihe goals of the experiment is to recover user’s private training image data, we first focus on the reconstruction of a single input image and the label from the gradients and model weights. In order to measure the effectiveness of data reconstruction attack, we measure Attack Success Rate, Attack iteration and the reconstruction error between each reconstructed image and it’s ground truth.

### 3.2. Overview of Single-Sample Reconstruction Attack System (SSRAS)

**Figure 2:**
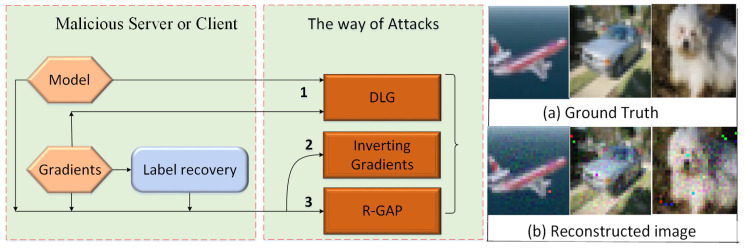
Single-Sample Reconstruction Attack System (SSRAS).

In this paper, we propose a Single-Sample Reconstruction Attack System. The overall framework of Single-Sample Reconstruction Attack System is illustrated in Figure 2.

Figure 2 shows the overview of Single-Sample Reconstruction Attack System. In Federated Learning, this security threat may come from: honest-but-curious server or malicious client. They can get gradients according to user’s private training image data, the architecture of neural network and the associated weight information. Based on the existing information, the server and client can reconstruct user’s private training image data. Let us consider a classification scenario.

Step 1, Label Restoration. In Federated Learning, the malicious server or client can make use of gradients according to the last fully connected layer to restore grouth truth label. It needs base condition that the previous layer before fully connected layer, has used non-negative activation functions, such as ReLU or sigmoid, which plays a key position.

Step 2, The way of Attacks. If the grouth truth label has been determined, we can use algorithm 2 or 3 to reconstruct the image. If the label cannot be determined, we also can use algorithm 1 to reconstruct the label, and then utilize R-GAP to recover user’s private training image data. In this way, it can extends the attack from the fully connected layer to the convolutional layer. Reconstructed image is better.

Step 3, Fidelity Regularization. In order to guide attack from gradients towards natural images, we adopt $R_{\mathrm{fidelity}\;}\left(\cdot \right)$ to the loss function to steer reconstructed image away from unrealistic image.

Step 4, Rank Analysis Index. Rank analysis Index provides an overall estimate of whether the data can be reconstructed.

In short, by using step 1 and 2, we can get a reconstructed image with some noise points. In order to make reconstructed image closer to the natural images, we can use step 3. The purpose of step 4 is to judge the security of the neural network according to the existing gradient and weight information.

#### 3.2.1. Label Restoration

Zhao et al. [10] extract the ground-truth from the shared gradient based on the last fully connected layer. It is single-sample batch as given gradient.

(18) $$c=i,\;\;s.t.\;\;\nabla W_{L}^{i^{T}}\cdot \nabla W_{L}^{j}\leq 0,\;\forall j\neq i$$

The negative gradient can be the index of the ground truth.

Multi-sample gradients are averaged over *k* images in a batch [23], Yin et al. [23] propose an algorithm for target class label recovery from given gradients.

(19) $$\nabla W_{m,n}^{\left(\mathrm{FC}\right)}=\underset{\mathrm{give}\;\mathrm{in}\;\nabla W^{\left(\mathrm{FC}\right)}}{\underset{︸}{\left(\frac{1}{k}\sum_{k}\nabla W_{m,k}^{\left(\mathrm{FC}\right)}\right)}}$$

There can utilize column minimum values to formulate the final label restoration algorithm for batch size *k*:

(20) $$\hat{y^{*}}=\arg\mathrm{sort}\left(\min_{m}\nabla_{W_{m,n}^{\left(\mathrm{FC}\right)}L}\left(x^{*},y^{*}\right)\right)\left[:K\right]$$

$x^{*}=\left[x_{1}^{*},x_{2}^{*},\ldots ,x_{k}^{*}\right]$ is the ground truth of batch size k. $y^{*}$ is the label corresponding to $x^{*}$.

Yin et al. [23] extend label Restoration algorithm from single-sample [10] to multisample in a batch. Note that, whether the label can be recovered from the gradient depends on the activation function of the last fully connected layer. It needs base condition that the previous layer before fully connected layer, has used non-negative activation function, such as ReLU or sigmoid, which plays a key position.

#### 3.2.2. The Way of Attacks

The relevant technologies used in this privacy attack system are Algorithm 1 (DLG) and Algorithm 2 (Inverting Gradients) belong to Optimization-Based Attack from Gradients. Algorithm 3 (R-GAP) belongs to Linear Equation Solver Attack.

DLG [9] adopts Formula (21) to recover the original input image $x^{\prime }{}^{*}$,$y^{\prime }{}^{*}$ from a transmitted gradient ∇W. The algorithm is described in Algorithm 1 in detail.

(21) $$\begin{array}{cc} x^{\prime }{}^{*},y^{\prime }{}^{*} & =\underset{x^{\prime },y^{\prime }}{\arg\min}{∥\nabla W^{\prime }-\nabla W∥}^{2}\\ \; & =\underset{x^{\prime },y^{\prime }}{\arg\min}{∥\frac{\partial \ell \left(F\left(x^{\prime },W\right),y^{\prime }\right)}{\partial W}-\nabla W∥}^{2} \end{array}$$

**Algorithm 1** DLG**Input:** F(x;W): Model; W: weights; ∇W: gradients of training data; **Result:** X, y private training data  1:/*Initialize dummy inputs and labels. */  2:$x_{1}^{\prime }\leftarrow N\left(0,1\right),y_{1}^{\prime }\leftarrow N\left(0,1\right)$  3:for i←1 to n do  4:/*Compute dummy gradients. */  5:$\nabla W_{t}\leftarrow \partial \ell \left(F\left(x_{t}^{\prime },W_{t}\right),y_{t}^{\prime }\right)/\partial W_{t}$  6:$D\leftarrow {∥\nabla W^{\prime }-\nabla W∥}^{2}+\alpha_{\ell_{2}}R_{\ell_{2}}\left(\hat{x}\right)$  7:/*Update data to match gradients.*/  8:$x_{i+1}^{\prime }\leftarrow x_{i}^{\prime }-\eta \nabla_{x_{i}^{\prime }}D,y_{i+1}^{\prime }\leftarrow y_{i}^{\prime }-\eta \nabla_{y_{i}^{\prime }}D$  9:end for10:return $x_{n+1}^{\prime }$, $y_{n+1}^{\prime }$11:end procedure


Inverting Gradients [11] proposes to use cosine distance. The specific form is shown in Formula (22). The algorithm is described in Algorithm 2 in detail.

(22) $$x^{\prime }{}^{*},y^{\prime }{}^{*}=\arg\min_{x\in [0,1]n}1-\frac{\langle \nabla_{\theta }L_{\theta }\left(x,y\right),\nabla_{\theta }L_{\theta }\left(x^{*},y\right)\rangle }{∥\nabla_{\theta }L_{\theta }\left(x,y\right)∥∥|\nabla_{\theta }L_{\theta }\left(x^{*},y\right)∥}+\alpha \mathrm{TV}\left(x\right)$$

**Algorithm 2** Inverting Gradients**Input:** F(x;W): Model; W: weights; ∇W: gradients of training data; **Result:** X, y private training data  /*Initialize dummy inputs and labels. */  2:$x_{1}^{\prime }\leftarrow N\left(0,1\right),y_{1}^{\prime }\leftarrow N\left(0,1\right)$for i←1 to n do  4:/*Compute dummy gradients. */$\nabla W_{t}\leftarrow \partial \ell \left(F\left(x_{t}^{\prime },W_{t}\right),y_{t}^{\prime }\right)/\partial W_{t}$  6:$D\leftarrow 1-\frac{\langle \nabla W,\nabla W^{\prime }\rangle }{∥\nabla W∥∥\nabla W^{\prime }∥}+\alpha_{TV}R_{TV}\left(\hat{x}\right)$/*Update data to match gradients.*/  8:$x_{i+1}^{\prime }\leftarrow x_{i}^{\prime }-\eta \nabla_{x_{i}^{\prime }}D,y_{i+1}^{\prime }\leftarrow y_{i}^{\prime }-\eta \nabla_{y_{i}^{\prime }}D$end for10:return $x_{n+1}^{\prime }$, $y_{n+1}^{\prime }$end procedure

R-GAP [22] breaks the gradient attack down to a recursive process of solving linear equations. They propose recursive procedure to recover data from gradient in deep neural networks. However, the gradient is aggregation gradient in CNNs. In order to effective analytic gradient attack for CNNs, there need peel off padding entries, and the stride should be appropriate, it need equal the size of convolutional kernel. Gradient constraints can be described as follows,

(23) $$K_{i}x_{i}=\mathrm{flatten}\left(\frac{\partial \ell }{\partial W_{i}}\right)$$

$x_{i}$ denotes the input in the ith layer and $K_{i}$ is a coefficient matrix containing all gradient constraints in the ith layer.

Weight constraints is shown in Formula (24), at the same time, it needs assumption that they know input of the subsequent layer.

(24) $$W_{i}x_{i}=O_{i};\;O_{i}\leftarrow f_{i}$$

$W_{i}$ is corresponding circulant matrix representing convolutional kernel, $\left|O_{i}\right|$ is weight constraints. The Formulas (23) and (24) can form matix A and B. The reconstructed user’s private training image data can be transformed into a matrix solution, $AX^{*}=B$. The condition of restoring user’s private training image is that the corresponding coefficient matrix A is equal to the number of entries.

#### 3.2.3. Fidelity Regularization

In order to guide attack from gradients towards natural images, we adopt $R_{\mathrm{fidelity}\;}\left(\cdot \right)$ to the loss function to steer reconstructed image away from unrealistic image [23]. $R_{\mathrm{TV}}$ and $R_{\ell_{2}}$ denote standard image priors, with scaling factors $\alpha_{\mathrm{tv}}$ and $\alpha_{\ell_{2}}$. The cost function of DLG is shown in Formula (25)

(25) $$D\leftarrow {∥\nabla W^{\prime }-\nabla W∥}^{2}+\alpha_{\ell_{2}}R_{\ell_{2}}\left(\hat{x}\right)$$

The cost function of Inverting Gradients is shown in Formula (26)

(26) $$D\leftarrow 1-\frac{\langle \nabla W,\nabla W^{\prime }\rangle }{∥\nabla W∥∥\nabla W^{\prime }∥}+\alpha_{TV}R_{TV}\left(\hat{x}\right)$$

R-GAP can make use of difference between reconstructed image, and adopt smooth version. The purpose of smooth version is to eliminate noise. There are two ways to do it in spatial domain or in frequency domain. Low—Pass filtering can be used to remove noise in spatial domain. While, the frequency domain can remove noise by removing high frequency components.

#### 3.2.4. Rank Analysis Index

Rank Analysis Index (RA-I) is used to measure the possible of whether the user’s raw image data can be reconstructed.

Fan et al. [19] analyze the Bias Attacks as a system of linear equations, and perform a rank analysis about network’s security. The matrix expression is as follows,

(27) $$B_{I^{.}}X^{*}=W_{I}$$

$B_{I}$ denotes the partial derivative of the loss function w.r.t.the model bias, $W_{I}$ denotes the partial derivative of the loss function w.r.t.the model weight parameter.

The condition of restoring user’s private training image is that the corresponding coefficient matrix $B_{I}$ is equal to the number of entries.

According to R-GAP algorithm, weight constraints can derived a new constraint, which is named virtual constraints $V_{i-1}$. The virtual constraints $V_{i-1}$ can be derived from the weight constraints of d-1 layer.

(28) $$W_{i-1}x_{i-1}=O_{i-1};\;O_{i}\leftarrow f_{i}$$

Split W, O into two parts,

(29) $$\left[\begin{array}{c} W_{i-1}^{+}\\ W_{i-1}^{-} \end{array}\right]x_{i-1}=\left[\begin{array}{c} O_{i-1}{}^{+}\\ O_{i-1}{}^{-} \end{array}\right]$$

(30) $$\begin{array}{cc} O_{i-1}{}^{+} & =I_{+}O\\ x_{i-1} & =W_{i-1}^{+}{}^{-1}I_{+}O\\ O_{i-1}{}^{-} & =I_{-}O\\ W_{i-1}{}^{-}x_{i-1} & =I_{-}O \end{array}$$

From Formula (28), we can get Formula (29) and Formula (30).

(31) $$\begin{array}{c} \left(W_{i-1}{}^{-1}W_{i-1}^{+}{}^{-1}I_{+}-I_{-}\right)O=0 \end{array}$$

Because of the activation function is the identity function, there is $O=x_{i-1}$. We can draw a conclusion as follows.

(32) $$\begin{array}{c} \left(W_{i-1}{}^{-1}W_{i-1}^{+}{}^{-1}I_{+}-I_{-}\right)x_{i-1}=0 \end{array}$$

(33) $$\begin{array}{cc} V_{i-1} & =W_{-}W_{+}^{-1}I_{+}-I_{-};V_{i-1}x_{i-1}=0 \end{array}$$

From Formula (33), virtual constrains $V_{i-1}$ can be derived from weights. Further, the virtual constrains of each layer can be derived, which based on the assumption that we know the output of the current layer.

Rank analysis includes gradient constraints $\left|W_{i}\right|$, weight constraints $\left|O_{i}\right|$ and virtual constraints $\left|V_{i}\right|$. It is named Rank Analysis Index (RA-I). All conclusions are based on assumption that the activation function is ReLU or LeakyRelu. The specific form of RA-I can be described by Formula (34).

(34) $$\;\text{RA-I}\;=\left\{\begin{array}{cc} \left|x_{i}\right|-\left|W_{i}\right|-\left|O_{i}\right|-\left|V_{i}\right|>0 & \;\mathrm{impossible}\;\\ \left|x_{i}\right|-\left|W_{i}\right|-\left|O_{i}\right|-\left|V_{i}\right|<0 & \;\mathrm{can}\; \end{array}\right.$$

$\left|x_{i}\right|$ represents the number of input entries in i-th layer, $\left|W_{i}\right|$ denotes gradient constraints in the ith layer, $\left|O_{i}\right|$ represents weight constraints in the ith layer, $\left|V_{i}\right|$ represents the number of virtual constraints in the ith layer.

RA−i>0, indicates that complete reconstruction cannot be performed. The larger the index, the worse the reconstruction quality. RA−i<0, indicates the ability to fully recover the input.

## 4. Improved R-GAP Alogrithm

**Figure 3:**
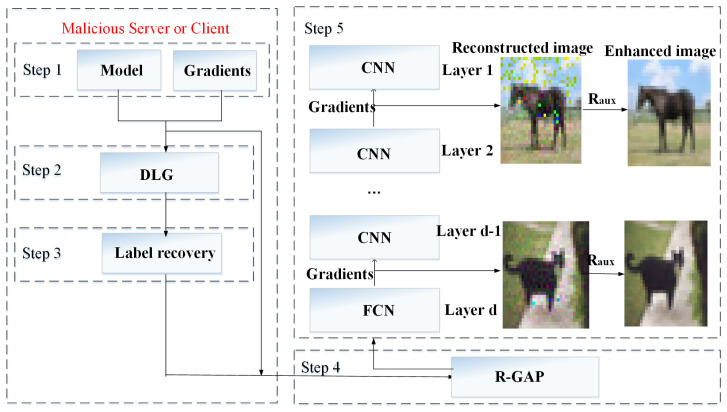
The framework of Improved R-GAP Alogrithm.

R-GAP works only if the label information is known. In this section, we propose Improved R-GAP Alogrithm, which can utlize DLG algorithm to derive ground truth. Further, it can extends the attack from the fully connected layer to the convolutional layer. The framework of Improved R-GAP Alogrithm is illustrated in Figure 3.

### 4.1. Advantage of Improved R-GAP Alogrithm

DLG algorithm [9] can recover private training data pixelwise accurate for images without label. However, this algorithm only can reconstruct image to fully connected layer. R-GAP [22] belongs to Linear Equation Solver Attacks. It works as well as or even better than Optimization-Based Attacks. This algorithm extends attack from the fully connected layers to CNNs, However, this algorithm works under the premise of label information is known. Based on the above analysis, we first choose DLG algorithm to determine label. Then, we choose R-GAP algorithm to recover user’s private training image data.

In summary, this Improved R-GAP Alogrithm can combine the advantage of the two algorithms. This improved algorithm can carried out image reconstruction regardless of whether the label can be determined.

### 4.2. Design about Improved R-GAP Alogrithm

Figure 3 shows design of the improved R-GAP algorithm. The algorithm consists of four parts:

Step 1, the malicious server or client get gradients according to user’s private training image data. At the same time, the architecture and weight information of neural network are known in advance.

Step 2, combining model and gradients, the malicious server or client utilize DLG algorithm to get reconstructed image.

Step 3, according to reconstructed image, the malicious server or client can infer category of the image.

Step 4, in case of knowing ground truth, the malicious server or client uses R-GAP algorithm to extend the attack from the fully connected layer to the convolutional layer.

In summay, the Improved R-GAP Alogrithm can represented as solving linear equations. The algorithm is described in Algorithm 3 in detail. The Formula (35) can form a matrix. The solving of the matrix can derive training image data.

(35) $$\begin{array}{cc} \; & \left\{\begin{array}{c} K_{i}x_{i-1}=\;\mathrm{flatten}\;\left(\frac{\partial \ell }{\partial W_{i}}\right)\\ W_{i}x_{i}=O_{i};\;O_{i}\leftarrow f_{i} \end{array}\right. \end{array}$$

**Algorithm 3** Improved R-GAP Alogrithm**Data: i: ith layer; $W_{i}$: weights; $\nabla W_{i}$: gradients;** **Result:** $X^{*}$  i←d to 1 **if** i=d **then**  3:  $\frac{\partial l}{\partial u}\cdot u=\nabla W_{d}W_{d}$    $\;\;u\leftarrow \frac{\partial l}{\partial u}\cdot u$; $K_{d}=\frac{\partial l}{\partial u}\cdot y$; $O_{i}=\frac{u}{y}$;**else**  6:  /* Derive $\sigma_{i}^{\prime }$ and $O_{i}^{\prime }$ from $f_{i}^{\prime }$. Note that $x_{i+1}=f_{i}.$*/  $\sigma_{i}^{\prime }\leftarrow x_{i+1}$; $Z_{i}\leftarrow x_{i+1}$  $K_{i}=\left(W_{i+1}^{⊤}\cdot K_{i+1}\right)⊙\sigma_{i}^{\prime }$  9:**end if**$\nabla W_{i}=flatten\nabla \left(W_{i}\right)$$A=\left[\begin{array}{l} K_{i}\\ W_{i} \end{array}\right]$; $B=\left[\begin{array}{l} \nabla W_{i}\\ O_{i} \end{array}\right]$12:$X^{*}=A^{-1}B$ **return** Outputs


## 5. Experiments and Results

In this section, we take the classification task as an example to verify the effect of the single-sample reconstruction attack system on two datasets: MNIST and CIFAR-10.

We first performed comparison of different gradient leakage attacks, for example DLG [9], Inverting Gradients [11], R-GAP [22], and our proposed attack system. Note that, when label can be recovered, Algorithm 3 adopts R-GAP, when label cannot be recovered, Algorithm 3 adopts Improved R-GAP Algorithm. Then, we showed the success of our proposed attack system compared with other algorithms. Finally, we showed that RA-I can predicts the risk of reconstruction.

### 5.1. Experiment Setup

Our analyses started from a case where the gradients are only calculated on one training sample (X,Y). We primarily focused on the CNN6 architecture for the classification task, which is following the settings in [22]. The choose of the activation functions is crucial, the last one is Sigmoid, the other are LeakyReLU. We used L-BFGS and Adam for optimization (learning rate 0.05). The attack terminates when the reconstruction learning is about to converge or the maximum number of attack iterations is reached.

### 5.2. Attack Effect and Cost Metrics

- Rank Analysis Index (RA-I). Rank analysis provides an overall estimate of whether the data can be reconstructed [22].
- Attack Success Rate (ASR). Attack Success Rate equals successfully reconstructed training data devide all the number of training data being attacked. ASR-content and ASR-label are two indicators about attack success rate on content and label, respectively [18,21].
- Attack iteration (Ai). It measures the max of attack iterations to converge [18].
- MSE. It shows the similarity between reconstructed image $f^{\prime }\left(i,j\right)$ and ground-truth image f(i,j). A smaller MSE means the more similar to the private ground truth [9,10].
  (36) $$MSE=\frac{1}{M\times N}\sum_{i=1}^{M}\sum_{j=1}^{N}{\left(f^{\prime }\left(i,j\right)-f\left(i,j\right)\right)}^{2}$$
  *M* and *N* represent the length and width of the image, respectively
- PSNR. It measures the ratio of effective information and noises in the reconstructed images, this indicator also is used in [11,21]. $x^{\prime }$ denotes reconstructed image and *x* denotes ground-truth image.
  (37) $$\mathrm{PSNR}\left(x,x^{\prime }\right)=-10\times \log_{10}^{\mathrm{MSE}\left(x,x^{\prime }\right)}$$
- SSIM. It measures the structural similarity between two images, which is used in [18]. The structural similarity ranges from 0 to 1. When two images are identical, the value of SSIM is equal to 1.
  (38) $$\mathrm{SSIM}\left(x,x^{\prime }\right)=\frac{\left(2\mu_{x}\mu_{x^{\prime }}+c_{1}\right)\left(2\sigma_{xx^{\prime }}+c_{2}\right)}{\left(\mu_{x}^{2}+\mu_{x^{\prime }}^{2}+c_{1}\right)\left(\sigma_{x}^{2}+\sigma_{x^{\prime }}^{2}+c_{2}\right)}$$
  where $u_{x}$ and $\mu_{x^{\prime }}$ denote the average of x and $x^{\prime }$, $\sigma_{x}^{2}$ and $\sigma_{x^{\prime }}^{2}$ denote the variance of x and $x^{\prime }$. $\sigma_{xx^{\prime }}$ denotes the covariance of x and $x^{\prime }$. $c_{1}={\left(k_{1}255\right)}^{2}$, $c_{2}={\left(k_{2}255\right)}^{2}$ are two variables to maintain stability. *k*1 = 0.01 and *k*2 = 0.03 are constants by default.

### 5.3. Results and Analysis

#### 5.3.1. Comparsion with Other Gradient Leakage Attacks

For the four algorithms DLG [9], Inverting Gradients [11], R-GAP [22], our proposed attack system, we performed experiments on the classification task over MNIST and CIFAR-10. The performance are shown in Table 2.

R-GAP [22] and Inverting Gradients [11], they can use the conclude that the value of ground-truth label $y_{1}^{*}$ can be derived from the sign of the gradients according to [10]. That is to say, R-GAP [22] and Inverting Gradients [11] work based on the assumption that the label can be analytically recovered. While DLG [9] can work, even if there is no ground-trouth label. Our proposed attack system combined the advantage of R-GAP [22] and DLG [9], this system can carried out image reconstruction regardless of whether the label can be determined. It can extends gradient inversion attack from fully connected layer with bias terms to attack fully connected layer and convolutional neural network with or without bias terms. When comparing attack iterates, we always provide DLG, Inverting Gradients, R-GAP and our proposed attack system the ground-truth label and let it recover the image only. DLG adopts L2 distance between the private training data and the dummy inputs. Inverting Gradients replaces the L2 distance function with cosine similarity.

From Table 2, according to attack iteration, our proposed attack system and R-GAP had lowest attack iterations on both content and label reconstruction respectively for all two datasets. We also observed that the algorithm of Inverting Gradients [11] can lead to high ASR-content compared with DLG [9], but at a great cost of attack iterations. R-GAP had a better quality of the reconstructed image than DLG [9] and Inverting Gradients [11]. Our attack system had a best performce on the high PSNR, SSIM and low MSE, because of adopting auxiliary regularization $R_{\mathrm{aux}\;}\left(\cdot \right)$.

**Figure 4:**
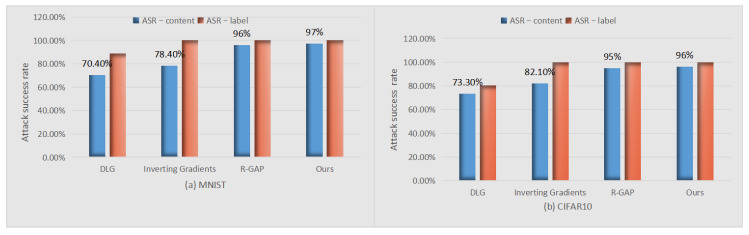
Comparison of gradient inversion attacks on two datasets.

From Figure 4, we saw our proposed attack system had a best performance on ASR-content. R-GAP [22] performed better than Inverting Gradients [11] on ASR-content. DLG [9] was worst on ASR-content. These concludes can be drawn under the assumption that the ground truth can be known. DLG [9] has its advantage, when there is no ground truth lable, it also can work.

**Figure 5:**
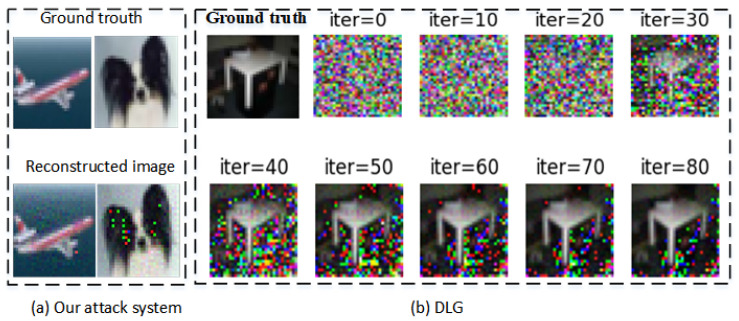
Ground truth and Reconstructed image by our attack system and DLG.

Figure 5 shows reconstructed image by our attack system and DLG algorithm. (a) is reconstructed image by our attack system. The upper layer is ground truth, The lower layer is reconstructed image. (b) is visualization showing from CIFAR-10 by DLG [9]. As the number of attack iterations increases, the reconstructed images are almost identical to ground truth, despite few negligible artifact pixels. But there need more time than our proposed attack system.

The activation function used in the neural network has a great influence in Gradient Inversion Attacks. In [18], the authors analyze the different characters of activation function, the most popular activation function ReLU can takes out the gradient information needed for attack, while sigmoid and tanh can transmit the gradient from layer to layer in almost lossless manner. In [21], they find the attacker can exploit the risky property of neural networks with ReLU, the single-sample can be reconstructed with low errors. We compared proposed attack system with R-GAP [22], Inverting Gradients [11] and DLG [9] on LeNet architecture. This architecture has been benchmarked in DLG [9], the statistical results are shown in Table 3. Our proposed attack system performed well on LeNet, however, we surprisingly found that by replacing the activation function Sigmoid with ReLU at fully conneted layer, the reconstruction of DlG algorithm and Inverting Gradients were hard to converge. The reason is the activation fuction can effect the security level of Net, which can reflect by RA-I. According to matrix A, the activation function sigmoid can lead to a higher virtual constrains condition numbers at each convolutional layer. It make our proposed attack system perform well. Yet, in the subsequent layer, the reconstruction error could be amplified, this make DLG [9] and Inverting Gradients [11] hard to converge. Our proposed attack system further adopted auxiliary regularization $R_{\mathrm{aux}\;}\left(\cdot \right)$ based on image fidelity to steer enhanced image away from unrealistic image, which was best of all.

From Table 4, experiments show MSE of the reconstruction over CIFAR10. The RA-I predicts the risk of reconstruction. It can be refelected by MSE. RA−I>0 indicates it is not possible to perform a complete reconstruction of the input. We can consider security level of the LeNet* can bear optimization-based attack. RA−I<0 implies the ability to fully recover the input. It means security level of the LeNet* can be attacked by R-GAP and our attack system. Because of adopting auxiliary regularization, our attack system had a best performce than other algorithms. Experiment results demonstrate Rank Analysis Index can estimate whether the data can be reconstructed. Further, it can provides an overall estimate of the security ot the neural network.

#### 5.3.2. Mitigation Strategies

In order to measure the effectiveness of the proposed algorithm (Improved R-GAP Alogrithm), we evaluated one attack mitigation strategy, which was gradient perturbation with additive noise. In this experiments, we adopted Gaussian noise. The Improved R-GAP Alogrithm was mitigated at cost of accuracy. As the Gaussian noise increases, the MSE becomes larger and larger. When we added sufficient Gaussian noise ($10\times 10^{-2}$), it made larger MSE. That showed poor quality of reconstruction attack, Table 5 provides the mitigation results.

## 6. Conclusions

In this paper, we make the first step towards a comprehensive survey about history of gradient leakage, and propose a privacy attack system, i.e., Single-Sample Reconstruction Attack System (SSRAS). This system can carried out image reconstruction regardless of whether the label can be determined. It can extends gradient inversion attack from fully connected layer with bias terms to attack fully connected layer and convolutional neural network with or without bias terms. We also propose Improved R-GAP Alogrithm, which can utilize DLG algorithm to derive ground truth. Furthermore, we introduce Rank Analysis Index (RA-I) to measure the possible of whether the user’s raw image data can be reconstructed.

We can see that it is not absolutely safety to exchanging model updates. If the relevant gradient protection measures are not used, personal privacy is at stake. Gradient contains a lot of useful information. There need a deeper understanding of gradient leakage attacks and privacy secure Federated Learning. We hope our study can arouse more research interests and efforts on the privacy properties of gradients and weights, in order to build more secure and privacy-preserving intelligent systems.

## Figures and Tables

**Table 1:** Representative attack algorithms for gradient inversion.

Method	Optimizer	Gradient Loss Function	Label Reconstruction Method	Categories of Attack
DLG [9]	L-BFGS	L2 norm	NO	Optimization-based
Inverting Gradients [11]	Adam	Cosine similarity	Know label	Optimization-based
R-GAP [22]	Adam	NO	Know label	Linear Equation Solver

**Table 2:** Comparison of Different Gradient Leakage Attacks.

	MNIST				CIFAR10			
	DLG [9]	Inverting [11]	R-GAP [22]	Ours	DLG [9]	Inverting [11]	R-GAP [22]	Ours
ASR-content	70.4%	78.4%	96%	97%	73.3%	82.1%	95%	96%
ASR-label	88.9%	100%	100%	100%	80.4%	100%	100%	100%
Ai	33	3216	1	1	80.2	6725	1	1
MSE	$3.7\times 10^{-5}$	$2.6\times 10^{-5}$	$1.9\times 10^{-5}$	$0.8\times 10^{-5}$	$0.8\times 10^{-4}$	$0.5\times 10^{-5}$	$0.12\times 10^{-5}$	$0.1\times 10^{-5}$
PSNR	33	36.3	40.1	40.6	34.2	36.8	40.8	41.8
SSIM	0.903	0.918	0.986	0.988	0.831	0.865	0.891	0.91

**Table 3:** Comparison of different activation function.

	MSE			
	DLG [9]	Inverting Gradients [11]	R-GAP [22]	Ours
LeNet	$5.2\times 10^{-2}$	$3.4\times 10^{-2}$	$0.25\times 10^{-4}$	$0.2\times 10^{-5}$
LeNet*	$0.6\times 10^{-4}$	$0.4\times 10^{-4}$	$0.44\times 10^{-4}$	$0.4\times 10^{-4}$

LeNet* is identical to LeNet but uses ReLU activation function instead of Sigmoid.

**Table 4:** RA-I predicts the risk of reconstruction.

	LeNet*			
	DLG [9]	Inverting [11]	R-GAP [22]	Ours
RA−I	405	105	−208	−208
MSE	$5.2\times 10^{-2}$	$3.2\times 10^{-3}$	$0.5\times 10^{-4}$	$0.4\times 10^{-4}$

LeNet* is identical to LeNet but uses ReLU activation function instead of Sigmoid.

**Table 5:** Mitigation strategies by Gaussian noise.

	CIFAR10			
Gaussian noise	$10\times 10^{-2}$	$10\times 10^{-3}$	$10\times 10^{-4}$	No noise
MSE	$3.0\times 10^{-1}$	$4.4\times 10^{-3}$	$6.9\times 10^{-4}$	$0.1\times 10^{-4}$

The values of Gaussian noise: means were zero, variance were different magnitude.

## Data Availability

This data can be found here: http://github.com/JunyiZhu-AI/R-GAP.

## Abbreviations

The following abbreviations are used in this manuscript:

FL	Federated Learning
SSRAS	Single-Sample Reconstruction Attack System (SSRAS)
RA-I	Rank Analysis Index
ASR	Attack Success Rate
LD	Linear dichroism
mGAN-AI	Multitask Generative Adversarial Network in artificial intelligence
CNN	Convolutional Neural Network
RNN	Recurrent Neural Network
DLG	Deep Leakage from Gradients
R-GAP	Recursive Gradient Attack on Privacy
SPN	Secret Polarization Network
CE	Cross-Entropy
